# Parental experiences of uncertainty following an abnormal fetal anomaly scan: Insights using Han’s taxonomy of uncertainty

**DOI:** 10.1002/jgc4.1311

**Published:** 2020-07-07

**Authors:** Jennifer Hammond, Jasmijn E. Klapwijk, Melissa Hill, Stina Lou, Kelly E. Ormond, Karin E.M. Diderich, Sam Riedijk, Celine Lewis

**Affiliations:** ^1^ North Thames Genomic Laboratory Hub Great Ormond Street Hospital for Children NHS Foundation Trust London UK; ^2^ Genetic and Genomic Medicine UCL Great Ormond Street Institute of Child Health London UK; ^3^ Department of Clinical Genetics Erasmus MC Rotterdam The Netherlands; ^4^ Center for Fetal Diagnostics Aarhus University Hospital Aarhus Denmark; ^5^ Department of Genetics and Stanford Center for Biomedical Ethics Stanford University School of Medicine Stanford CA USA; ^6^ Population, Policy and Practice Department UCL Great Ormond Street Institute of Child Health London UK

**Keywords:** communication, fetal anomaly, parents, prenatal diagnosis, uncertainty

## Abstract

For a number of prospective parents, uncertainty during pregnancy starts when an anomaly is found during a routine fetal anomaly scan. This may be followed by numerous tests to determine the etiology and nature of the anomaly. In this study, we aimed to understand how prospective parents perceive and manage uncertainty after being confronted with a structural anomaly during their routine ultrasound. Han's taxonomy of uncertainty was used as a framework to identify and understand the different types of uncertainty experienced. Interviews were held in the UK (*n* = 8 women and *n* = 1 male partner) and in the Netherlands (*n* = 7 women) with participants who had experienced uncertainty in their pregnancy after a fetal scan. Data were analyzed using thematic analysis, and the uncertainties experienced by parents were mapped against the dimensions of the Han taxonomy (sources, issues, and locus). Participants' experience of uncertainty was relevant to all dimensions and subcategories of the Han taxonomy, showing its applicability in the prenatal setting. Sources of uncertainty included receiving probabilistic or ambiguous information about the anomaly, or information that was complex and challenging to understand. Issues of uncertainty included were those that were scientific—such as a probable diagnosis with no further information, personal—such as the emotional impact of uncertainty, and practical—such as limited information about medical procedures and practical aspects of care. Additionally, participants described what helped them to manage uncertainty. This included active coping strategies such as searching for information on the Internet, external coping resources such as seeking social support, and internal coping resources such as using positivity and hope. Several recommendations for the healthcare professional to minimize uncertainty and help the patient deal with uncertainty have been proposed based on these findings.

## 1 INTRODUCTION

Antenatal screening for fetal structural anomalies has become part of routine pregnancy care in most Western countries since its introduction in the 1970s. Ultrasound scans play an important role in fetal surveillance during the course of pregnancy. These scans are also important in the monitoring and management of high‐risk pregnancies, to reduce obstetric interventions and the risk of perinatal deaths (Alfirevic, Stampalija, & Dowswell, 2017; Edvardsson, Small, Persson, Lalos, & Mogren, 2014). In the majority of cases, routine ultrasound provides prospective parents with the opportunity for reassurance about the health and development of their baby (Garcia et al., 2002). However, in up to 5% of pregnancies, a structural anomaly will be detected (Best et al., 2017; Boyd et al., 2011). This can be frightening and upsetting for parents (Aite et al., 2011; Skreden et al., 2010) and can result in uncertainty for both parents and healthcare professionals (HCP) if the findings are ambiguous (Asplin, Wessel, Marions, & Georgsson Öhman, 2012; Resnik et al., 2018). Feelings of uncertainty can be further amplified when parents are not prepared for anomalies to be detected (Garcia et al., 2002), or if the information given about the capabilities and limitations of the ultrasound scan is inadequate (Asplin et al., 2012; Crang Svalenius, Dykes, & Jörgensen, 1996; Eurenius, Axelsson, Gällstedt‐Fransson, & Sjöden, 1997; Lalor & Devane, 2007). When an anomaly is detected on an ultrasound, parents may be offered invasive diagnostic testing, to allow target genetic tests, chromosomal microarray analysis (CMA) (Wapner et al., 2012), or, increasingly, exome sequencing (ES) (Mone, Quinlan‐Jones, & Kilby, 2018). While these diagnostic tests may lead to a clinical diagnosis, some parents will receive inconclusive results, variants of uncertain significance (VUS), or no diagnostic information.

Uncertainty can be described as a state of having ‘imperfect or unknown information’ (Gollust et al., 2012). Currently, the literature on uncertainty within the field of prenatal testing concentrates on uncertainty following ultrasound or diagnostic testing, where a lack of information can emotionally affect parents (Wou et al., 2018) and complicate pregnancy decision‐making (Richardson & Ormond, 2017). In a prenatal testing setting, uncertainty may arise in a number of ways. For example, parents may be given a diagnosis with an uncertain prognosis; an unexpected diagnosis that may or may not have been related to the testing indication; a VUS for which no or limited prognostic information is available, or a result where ‘no meaningful information is found’ and a genetic cause of the fetal abnormality cannot be given (Richardson & Ormond, 2017). This can lead to many parents experiencing emotional turmoil as they do not have concrete information on which to base pregnancy‐related decisions such as whether to undergo invasive testing, which can carry a small but significant risk of miscarriage (Quinlan‐Jones, Hillman, Kilby, & Greenfield, 2017; Salomon, Sotiriadis, Wulff, Odibo, & Akolekar, 2019), and whether to continue the pregnancy or have a termination of pregnancy (Sommerseth & Sundby, 2010). Given that most parents have entered into prenatal testing expecting a diagnostic test that provides clarity, rather than uncertainty (Richardson & Ormond, 2017), this change in their expectations further complicates further decision‐making. For those that continue with their pregnancy, anxiety can persist long after the birth if an explanation for the anomaly is not found, with concerns that history may repeat itself in future pregnancies (Garcia et al., 2002).

One approach to understanding uncertainty in healthcare settings is the conceptual taxonomy proposed by Han et al. which includes three major dimensions to describe medical uncertainty: *source*, *issue,* and *locus* (Han, Klein, & Arora, 2011) (Table 1). The value of the taxonomy is that it facilitates differentiation between the many types, sources, and manifestations of uncertainty within healthcare settings (Han et al., 2011), and furthermore, allows for the investigation of uncertainty from multiple perspectives (Makhnoon, Shirts, & Bowen, 2019). Han's taxonomy of uncertainty has been used in a number of different healthcare settings. For example, Pickles et al applied this taxonomy to outline the different sources of uncertainty General Practitioners described encountering in relation to prostate cancer screening in adults (Pickles, Carter, Rychetnik, McCaffery, & Entwistle, 2016).

**TABLE 1:** Dimensions of Han's Taxonomy as paraphrased from Makhnoon et al. (2019)

Source	Refers to the cause of a given uncertainty and is subdivided into: *Probability* which refers to the fundamental indeterminacy or randomness of future outcome *Ambiguity,* which refers to a lack of reliability, credibility, or adequacy of risk estimates C*omplexity* which refers to features of risk information that make it difficult to understand, such as conditional probabilities or multiplicity in risk factors
Issue	Refers to the implications of uncertainty which can depend on what information is uncertain and is subdivided into: *Scientific* (diagnostic, prognostic, causal, or therapeutic) *Personal* (psychosocial and existential issues) *Practical* (lack of knowledge about the structures and processes of health care)
Locus	Describes within whom the uncertainty lies and can include the following: Patients Clinicians Researchers Health policy makers

Han's taxonomy has also been applied in studies of clinical genomic sequencing, using a version of the taxonomy specifically developed for this purpose (Han et al., 2017). The three major dimensions (source, issue, and locus) remain the same, but additional layers further discriminate uncertainties relevant to variant findings in genomic sequencing.

Makhnoon et al used the taxonomy to describe the sources and issues of VUS‐related uncertainty from the patient perspective (Makhnoon et al., 2019), and similarly, Park et al. applied the taxonomy to understand genetic counselors perceptions of uncertainty in pre‐test counseling (Park, Zayhowski, Newson, & Ormond, 2019).

For many parents in the prenatal setting, their experience of uncertainty begins with the detection of an anomaly at a routine ultrasound examination. As far as we are aware, no study has applied Han's taxonomy to gain insight into the parent's experience of uncertainty following prenatal ultrasound examination, and how the uncertainty is managed. Using this taxonomy may lead to improved understanding of the different aspects of uncertainty and their impact in a prenatal setting and provide guidance for ways to support parents in managing their uncertainty. This may help both parents and clinicians establish realistic expectations of testing processes and outcomes. Here we describe qualitative interviews with parents from the United Kingdom (UK) and the Netherlands (NL) who have experienced uncertainty during their pregnancy and map these findings onto Han's taxonomy of uncertainty.

## 2 METHODS

### 2.1 Study design

This is a qualitative study using semi‐structured interviews with parents who had experienced uncertainty during their pregnancy following a routine prenatal ultrasound examination. This work is part of a larger international study; the primary aim of the interviews was to inform the development of a discrete choice experiment to look at parents’ preferences and tolerances for uncertainty in prenatal testing. During these interviews, parents discussed the experiences of receiving uncertain results and how they managed their uncertainty. We undertook a qualitative analysis of the dataset focusing on the issue of uncertainty. We used the medical taxonomy of uncertainties by Han et al. (2011) to analyze the data through its three major dimensions (sources, issues, and locus).

### 2.2 Study setting

Interviews were conducted in the UK and NL. These two countries routinely offer the fetal anomaly scan, with an uptake of over 90% (Gitsels‐van der Wal et al., 2014; Ward & Soothill, 2011). In the UK, there is an agreed policy in place for screening fetal anomalies outlined by the Department of Health through the UK National Screening Committee (UK NSC) (Boyd et al., 2011). NICE guidelines state that ultrasound screening for fetal anomalies should be routinely offered normally between 18 weeks and 0 days and 20 weeks and 6 days (National Institute for Health & Care Excellence, 2008). The NHS Fetal Anomaly Screening Programme (FASP) lists nine structural congenital anomalies or groups of anomalies and two chromosome anomalies that women should be screened for at the anomaly scan (Public Health England, 2018).

In the NL, patients are part of a compulsory health insurance scheme (Schäfer et al., 2010), with some elements of prenatal testing covered under their policy. Ultrasound screening for physical abnormalities (anomaly scan) in the fetus is covered under this insurance policy. The Dutch National Institute for Public Health and the Environment (RIVM) (Dutch National Institute for Public Health & the Environment., 2019) and guidelines developed by the Dutch Association for Obstetrics and Gynaecology (NVOG) (Nederlandse Vereniging voor Obstetrie en Gynaecologie., 2019) outline that the anomaly scan is routinely offered, after counseling, between 18 weeks and 0 days and 21 weeks and 0 days, but ideally in week 19.

### 2.3 Recruitment

To meet the inclusion criteria, participants had to be over 18 years of age and had a structural anomaly identified on ultrasound without a clear diagnosis and/or prognosis. Participants were excluded if the uncertainty was related to receiving a high‐risk Down syndrome screening result, as this study was focused on rarer genetic anomalies. Participants had to be able to speak the local language where interviews were conducted. We did not stipulate a time limit for when the uncertainty was detected during the ultrasound scan, for example, more than 5 years ago, as we did not want to limit the number of potential participants.

In the UK, prospective participants were recruited through an advertisement placed on the Facebook page of Antenatal Results and Choices (ARC), a charity that supports parents through antenatal testing. Interested participants who fit the inclusion criteria were asked to contact the research team and provide more information about their uncertain result so that the research team could assess their suitability to take part in the study. Potential participants were emailed an information sheet, and a date and time to conduct a telephone interview was arranged with a researcher (JH or CL). Two participants (a couple) were invited to participate by the research team as they were participants in a separate interview study where a significant part of their experiences described in the interview was focused on uncertainty in the prenatal setting. They were invited to take part in this study via email and sent a participant information sheet. Three potential participants who contacted the research team were excluded due to a Down syndrome diagnosis obtained through amniocentesis, and two potential participants did not take part due to the recent loss of a baby and concerns regarding participant distress. In the NL, potential participants were recruited from a cohort of patients who had undergone prenatal ES following an abnormal fetal anomaly scan. After they received their ES results, potential participants were approached by telephone by their clinical geneticist at Erasmus Medical Centre to take part in an interview. Those that agreed to participate were then contacted by a researcher (JEK) at Erasmus Medical Centre to arrange a date and time to conduct the telephone interview and were sent a participant information letter. Interviews in the UK and the NL were conducted between February and June 2019.

### 2.4 Data collection

A topic guide (Supporting information S1) was developed in English in collaboration with an advisory team from the UK, the NL, Denmark, and the USA, comprising genetic counselors, social scientists, a patient advocate, a geneticist, and a health psychologist. As interviews were conducted as part of a larger international study to inform the development of a discrete choice experiment, we are only presenting data from the interviews that is relevant to this study which include the following: parents experience of receiving uncertain results; what was uncertain about the results, how the results were explained, the emotional and clinical impact of the uncertain results, how they managed the uncertainty, and decision‐making based on uncertain results. The topic guide was translated into Dutch for interviews conducted in NL. All interviews in the UK and the NL were conducted via telephone. Consent for interviews in both countries was obtained prior to starting the interview. Participants were given the opportunity to ask questions before providing consent. In the UK, interviews were conducted by two female social scientists (CL and JH). In the NL, data were collected by a female researcher who was completing a Masters’ degree at Erasmus University Rotterdam (JEK). Interviews took place between February and June 2019, and participants in the UK were offered a £10 gift voucher for their time. All interviews were recorded using a digital voice recorder and transcribed verbatim. Dutch recordings were transcribed by JEK. Dutch transcriptions were translated into English using Google Translate and were checked by JEK, who is a native Dutch speaker.

### 2.5 Data analysis and validation

Data analysis was facilitated using NVivo version 12 (QSR International) and analyzed thematically (Braun & Clarke, 2006) using an abductive approach, which engages in a two‐way dialogue between data and theory (Hiles, 2014). This approach was suitable for the qualitative analysis of this study, where we would be drawing together constructs from Han's taxonomy to explain and apply context and meaning to the data obtained (Hiles, 2014). Data were collected and analyzed concurrently, and data collection ceased when content saturation had been reached and no new themes were identified from the interviews. The transcripts from UK and Dutch participants were treated as one dataset, with the sample considered too small and diverse for meaningful comparisons. Four UK transcripts were independently coded by JH and JEK to create a coding framework. Once an initial coding framework was developed, the Han taxonomy was applied and codes further refined to see how they mapped onto the three overarching dimensions of the taxonomy (source, issues, and locus). Three UK and three Dutch transcripts were then coded again following the dimensions of the taxonomy by JH and CL. As parents also spoke of how they managed the uncertainty, an additional theme focusing on management strategies was added to the coding framework. The application of the taxonomy to the dataset was discussed at various stages between JH, CL, and MH to make decisions about where individual codes and themes fit within the taxonomy, and at each stage, minor changes to the coding framework were made. Once a coding framework using the taxonomy had been finalized, the remaining transcripts were coded by JH and JEK. The findings of the analysis, including the application of the taxonomy to the dataset, were discussed by all members of the research team.

## 3 RESULTS

### 3.1 Participants

Sixteen parents completed interviews; nine from the UK, and seven from the NL. All but one was female, and most were bachelor degree educated (15/16). Eleven women had undergone invasive testing following an ultrasound where an abnormality was found. Of these, seven Dutch women had ES. No UK women had ES. The types of uncertainty that were experienced at the ultrasound included structural anomalies such as a growth in the stomach, missing or malformed limbs, cardiac anomalies, fetal megacystis, or a smaller than expected fetus. In one case, a potential but unconfirmed diagnosis of Dandy–Walker syndrome and Joubert syndrome was given. Nine women reported choosing to have a termination of pregnancy (TOP) (Table 2). Uncertainties had occurred between one and 11 years ago. Interviews lasted between 35 and 61 min, with an average interview time of 49 min.

**TABLE 2:** Characteristics of interview participants

	UK *n* = 9 (*n* = 8 pregnancies)	the Netherlands *n* = 7
Age range [mean]	29–44 [36.1]	26–35 [30.4]
Gender		
Male	1	0
Female	8	7
Ethnicity		
Caucasian	6	Data unavailable
Asian/Asian British	3	Data unavailable
Black/Black British	0	Data unavailable
Other	0	Data unavailable
Religion		
Muslim	2	0
Christian	3	0
Catholic	0	1
Other	0	0
None	4	6
Education level		
High school	1	0
Bachelor's degree	8	7
Had invasive testing?		
Yes	4	7
No	4	0
Terminated pregnancy where uncertainty arose?		
Yes	5	4
No	3	3
How long ago uncertainty was experienced		
<1 year	2	5
1–2 years ago	3	2
3+ years ago	4	0

### 3.2 Key themes

We identified three overarching themes. Two themes matched the dimensions of Han's taxonomy of uncertainty; primarily ‘*sources of uncertainty’* and ‘*issues related to uncertainty’*. The third theme focused on ‘*managing uncertainty*’. Han's ‘Locus’ of uncertainty was not expanded on as a theme, as the locus in all cases here is the parent experiencing the uncertainty. The findings reflect parents’ experience of an uncertain result identified during the fetal anomaly scan, including the experience of uncertainty as parents underwent further testing and/or made decisions about TOP.

#### 3.2.1 Sources of uncertainty

##### Probability (a fundamental indeterminacy or randomness of future outcome)

Following the anomaly scan, some parents reported receiving information related to risk or chance of a particular prognosis, including whether the condition was genetic. For example, one Dutch parent, whose baby was found to have a thickened neck fold and proceeded with an amniocentesis, explained being told that she might have a ‘50% chance of a child with a [genetic] disorder and a 50% chance of a healthy child’ (NL7, female, third child, pregnant at interview). In some cases, chance was described using verbal descriptors rather than numerical risks. For example, one UK parent, whose baby was found to have an abnormal growth in the stomach explained ‘They said the baby *could be* disabled, it *could be* a chromosome problem’. (UK5, female, first child, continued pregnancy). Another example of probabilities that HCPs spoke about related to recurrence risk. One Dutch parent recalled being informed that there was a 25% chance of a possible recurrence of the defect detected (NL3, female, second child, chose TOP). Probability was also used to present the chance of miscarriage following an invasive procedure. As one UK parent, whose ultrasound scan indicated that the baby's brain had not developed properly explained, ‘my options at that time were to have a CVS [chorionic villus sampling] test the next day but with a 3% chance of miscarriage, and I just thought that was too high at that time’. (UK3, woman, first child, chose TOP). For some couples, any risk of miscarriage was considered too high, particularly in light of feeling protective toward the pregnancy.

Finally, probability was also used to discuss the likelihood of the baby surviving, which created uncertainty that was especially difficult for parents to process. Uncertainty about the baby's chances of survival could be a major source of anxiety during pregnancy. An example was given by one Dutch parent who described being informed that her unborn child had a ‘60% chance of survival’. This uncertainty over death and survival had such a profound effect on how she felt about her pregnancy, that she described focusing on the ‘40% [the baby] will not survive’ rather than the higher figure associated with survival (NL4, female, second child, pregnant at interview).

##### Ambiguity (lack of reliability, credibility, or adequacy of risk estimates)

Parents described two types of ambiguity that they experienced. The first related to a diagnosis or information about the condition itself being unavailable. For example, one Dutch parent explained that ‘the obstetrician said that she had seen the abnormality and that she did not know what it meant’ (NL3, female, second child, chose TOP). Additional diagnostic testing, such as CMA or ES did not always provide parents with further information or clarity. One UK parent, whose baby was found to have a larger than normal head and an extra digit on one of the hands, described how HCPs were not able to offer any further information other than a ‘clinical suspicion that this was probably something genetic’ (UK1, partner, first child, continued pregnancy).

A second type of ambiguity related to the ‘imprecision’ of the available information. Examples of this included the prognosis for the baby including whether the pregnancy would be viable and survive to full‐term, and imprecision related to conditions associated with incomplete penetrance or variable expressivity. One Dutch parent, who was informed that the baby had a congenital heart defect, described being given a range of possible outcomes from ‘very intense and which linger’ to other children having ‘better outcomes’ (NL4, female, pregnant with first child at interview, continued with pregnancy).

##### Complexity (features of risk information that make it difficult to understand)

How the identified anomaly was explained could be difficult for parents to understand. This was articulated by one Dutch parent who described how her HCP ‘had to explain things a bit more often’ to her (NL3, female, second child, chose TOP). Similarly, one UK parent, who had been told that the measurements for their baby did not seem quite right and was given a probable diagnosis of triploidy, described having to ‘go and research’ the unfamiliar terminology associated with the probable diagnosis she was given (UK8, female, second child, chose TOP). The language used to describe procedures could also be medicalized and complex. This was reflected on by one UK parent who was told by her HCP that a termination after 24 weeks would involve a ‘feticide’, further stating: ‘I didn't know what that means and if you Google it, it's not nice, is it?’ (UK6, female, first child, chose TOP).

Finally, complexity was also found to occur when more than one possible diagnosis was discussed at the same time, which could be overwhelming for parents to take in. This was highlighted by one partner who commented ‘It was floated quite early on that this could be what it ended up turning out to be—Joubert's syndrome. There were some other things that were floated as well which is a lot more serious… So that was a bit overwhelming as well.’ (UK1, partner, first child, continued pregnancy).

#### 3.2.2 Issues related to uncertainty

##### Scientific (diagnostic, prognostic, causal, or therapeutic)

Following the identification of an anomaly, some parents were given potential, unconfirmed diagnoses (*diagnostic* issue). For example, the UK parent whose baby was given a potential diagnosis of Joubert's syndrome, described this potential diagnosis was given with caveats such as ‘“mights,” “buts” and “maybes”’ (UK2, female, first child, continued pregnancy).

Additionally, there could be frustration that despite undergoing further diagnostic tests, the results did not necessarily provide any further clarity about potential treatments (*therapeutic* issue) as described by one partner who commented that there was ‘not much that can be offered by way of intervention’ for his unborn child (UK1, partner, first child, continued pregnancy).

All parents would have questions about the etiology of the anomaly (*causal* issue). One UK parent described having lingering questions regarding the cause of the anomaly after she had ended the pregnancy, as no further information could be given saying: ‘we don't know [what caused it], it might have been [genetic]’ (UK8, woman, second child chose TOP). In nine cases, this was not the first pregnancy, and the detection of an anomaly raised questions about how it happened alongside comparisons against the ‘normal’ first pregnancy previously experienced: ‘You're thinking well, you know, why did this one fail? Why didn't this one survive?’ (UK8, female, second child, chose TOP).

Finally, parents who underwent additional non‐invasive procedures, such as further ultrasound scans or fetal MRI scans, also experienced *prognostic* issues related to uncertainty. For example, one UK parent explained that despite a possible diagnosis for her unborn child, there was no certainty that her baby would survive the delivery: ‘There was still the ‘but she's so small, how is she ever going to survive the delivery’ (UK7, female, second child, continued pregnancy).

##### Personal (psychosocial and existential issues)

Discovering that their unborn child had a fetal anomaly came as a shock and affected parents emotionally. One Dutch parent described the emotional impact, recounting ‘it makes you very sad because you don't expect it’ (NL8, female, first child, chose TOP). One UK parent spoke of the loneliness felt during this time, and not knowing ‘what to do, how to feel’ (UK4, female, second child, chose TOP). Some parents were found to self‐blame, believing that they had caused the anomaly identified on the scan, with one parent questioning ‘Did I do something wrong? Or has that been a defect of nature?’ (NL8, female, first child, chose TOP). Uncertainty could also affect the ‘joy’ of pregnancy. One UK parent reflected on the grief she felt that an enjoyable pregnancy was ‘taken away’ from her (UK5, female, first child, continued pregnancy), while another UK parent explained that they ‘didn't actually buy anything for the baby’ (UK1, partner, first child continued pregnancy) because as they were unable to be certain what the prognosis would be for their child, and whether she would survive to term.

Decision‐making around TOP was particularly challenging, and parents spoke about having to consider and potentially proceed with TOP. One UK parent spoke of her internal debate regarding termination, which was focused around the uncertainty of whether her child would be born with a single malformation or a syndrome ‘I was thinking, OK, so if it's a growth, we could just have it removed and the baby will be OK, but I don't know if the baby will be born and it's got some sort of syndrome and he is disabled’ (UK5, female, first child, continued pregnancy). For participants who opted for TOP, there was guilt over their decision. One UK parent expressed that ‘maybe I should have had an amniocentesis’ (UK3, female, first child, chose TOP), despite the ultrasound showing severe malformations.

The uncertainty surrounding the etiology of the anomaly also caused concern for the potential recurrence in future pregnancies: ‘There is another uncertainty in its place. That things may go wrong with a subsequent pregnancy and whether a new syndrome will arise or not during the next pregnancy’ (NL8, woman, first child, chose TOP). For some parents, this experience of uncertainty was too much and appeared to influence their decision about future pregnancies, with another Dutch parent explaining ‘I don't want any more children. And maybe it's because of the stress I've had because of the uncertainties. I just never want to experience it again’ (NL1, female, pregnant at interview with first child, continued with pregnancy).

Anxiety could persist long after the birth, especially when no further diagnostic information to explain the anomaly was found. In these circumstances, parents remained vigilant to see if signs or symptoms that could be related to the anomaly would reoccur. One UK parent, whose son was found to have a mass on his stomach during the ultrasound scan which later disappeared, explained how ‘for the first two years of his life, whenever he had tummy problems I thought "oh maybe it's this mass again"’ (UK5, female, first child, continued pregnancy).

##### Practical (lack of knowledge about the structures of health care and processes of health care)

Both UK and Dutch parents described uncertainties related to the systems and structures within their healthcare system. One UK parent who had opted for further testing following her abnormal ultrasound result, described having to ‘chase basically three hospitals’ about what would be happening next, in particular to see which hospital she had to attend, what type of test she was having, and when it would be taking place (UK4, female, second child, chose TOP).

Clinical appointments that were arranged quickly, or where little information was provided about the reason for the appointment, could also create additional uncertainty for parents. One UK parent described feeling inadequately prepared for what might be discussed at a follow‐up appointment, describing that she was ‘literally catapulted from a slight heart defect to some serious, serious issues’ (UK6, female, first child, chose TOP). Additionally, one Dutch parent explained that it was ‘only later when I had been to [name of] hospital it became clear why everything had to be arranged so quickly’ (NL5, female, first child, chose TOP).

A number of parents also described procedural uncertainty related to the process and timelines of decision‐making around TOP. One UK parent recounted not knowing how quickly she would have to make a decision about ending her pregnancy, being told by HCPs ‘to ring us tomorrow morning with your decision because we've got to get you booked in to get it done’ (UK6, female, first child, chose TOP).

Parents also experienced procedural uncertainties regarding the process following the birth of their child. In some cases, this could leave parents feeling apprehensive about the hospital they would be attending. This was explained by one Dutch parent who had planned a tour of the intensive care unit (ICU) during the last few weeks of her pregnancy to see where her baby would be transferred following the birth. However, when arriving at the unit, she found that the staff were unaware of their planned visit, explaining, ‘we got there and they said ‘oh, that was not passed on to us at all, that is really bad’ and ‘uhh…which unit then?’ I didn't know and they didn't know’ (NL4, female, pregnant at interview with first child, continued pregnancy). This made her feel uneasy about the ward and having to ‘put my child there later’. One UK parent spoke of being unaware of the processes should their baby not survive the birth, explaining: ‘I remember the day before just Googling actually what practically happens if a baby doesn't make it in terms of the funeral arrangements and everything’ (UK1, partner, first child, continued with pregnancy).

#### 3.2.3 Managing uncertainty

##### Parent strategies

As it was not always possible for uncertainty to be resolved, parents adopted a range of strategies to help manage different aspects of uncertainty.

###### Active coping strategies: Information seeking

Active coping strategies, such as information seeking, could be instrumental in helping parents to manage the *source* of uncertainty, particularly if the information given, for example, particular words or possible diagnoses, were *complex*. One Dutch parent, given a probable congenital heart defect diagnosis, explained that searching the Internet helped her to understand the diagnosis and the conversation she had with her HCP during the appointment ‘a little better’ (NL4, female, pregnant at interview with first child, continued with pregnancy). Another UK parent reflected on the importance of using the Internet to help her understand the discussions regarding the anomaly stating, ‘If it hadn't have been for the internet, I have no idea where I would have got any of that information from’. (UK8, female, second child, chose TOP).

Searching the Internet, however, could also be linked to anxiety with some parents expressing fear of what they might find. For example, one UK parent explained that after her appointment she ‘found a few [search results] that could have been possible a diagnosis’ but that ‘they weren't good either’ (UK5, female, first child, continued pregnancy). It could also be difficult to know whether information found online was trustworthy, with one UK parent explaining that you're relying on ‘whatever you can find on the internet being right’ (UK8, female, second child, chose TOP).

###### External coping resources: Social and psychological emotional support

External coping resources such as seeking social support were helpful to many in coping with *issues* of uncertainty, both *personal* and *practical*. Family, friends, and partners were often a source of support for parents going through this period of uncertainty: ‘We have had a lot of support in that regard from friends and family and everyone involved with us. They made it clear that they were there and that they wanted to help’. (NL5, female, first child, chose TOP). Additionally, support from outside a parent's close social group could be beneficial. Social media platforms, such as Facebook, helped parents to connect with other parents or families who had gone through similar experiences. Through this bond, parents were able to share their own experiences, relate to and learn from others. One Dutch parent commented: ‘there was a platform on Facebook which was really helpful for people with children, or patients who have CHD [congenital heart disease]’ (NL4, female, pregnant at interview with first child, continued pregnancy).

A number of UK participants spoke of being directed to support groups or charities, such as ARC or SANDS (the Stillbirth and Neonatal Death charity), who supported them. While these groups were reported as being helpful, some UK parents felt that they were directed to these support groups too late, with one parent explaining ‘I found out about them (ARC) after, but had I maybe been signposted to them when I went to meet the midwife to talk about ending the pregnancy, it would have been nice. That could have been helpful’. (UK3, female, first child, chose TOP).

Dutch parents spoke of being referred to psychologists to help them to manage the uncertainty. Those who chose to see a psychologist reported feeling that they had the support needed and felt understood by their clinician. However, some parents did not feel that they needed psychological support at the time with one commenting ‘we could always rely on a psychologist, but we deliberately didn't do that because we knew very well where we stood about the choice we made’ (NL3, female, second child, chose TOP). In contrast, no UK parent in our study discussed having access to or a referral to psychological support. One UK parent explained how she ‘didn't have any counseling, didn't have anyone to speak to, I had to weigh it all up myself’ (UK5, female, first child, continued pregnancy) and another stated ‘any kind of mental health support would have been good’ (UK6, female, first child, chose TOP). Despite this, there were examples of HCPs, such as midwives or the consultant, providing emotional support. One UK parent recalled the emotional support given to her by her midwife and consultant, describing how she felt supported in their decision to terminate their pregnancy explaining ‘I felt like they all made us feel very supported… she knew that was a really hard decision to make. She knew that we felt absolutely rotten in that moment’ (UK3, female, first child, chose TOP).

###### Internal emotion coping resources—positivity and hope

Parents also demonstrated uses of internal emotional coping resources to manage the *personal* impact of uncertainty. Some parents reported trying to remain ‘hopeful’ for a better outcome, despite the lack of clarity around the uncertainty: ‘you do not know what is going on and but you still have hope that it is not too bad’ (NL3, female, second child, chose TOP). In the midst of the uncertainty, one UK parent described that having hope ‘kept me going’ through the pregnancy (UK5, female, first child, continued pregnancy). Avoidance strategies were also used; one UK participant described consciously deciding to defer thinking about the potential outcomes, saying that she was not going to ‘deal with this just yet, until I really have to’ (UK2, female, first child, continued pregnancy).

There were examples of using positive reinterpretation and acceptance as a way to cope with uncertainty and decisions about TOP. For example, one UK parent described that despite having to choose whether to end her pregnancy, she described herself as ‘lucky’ that the decision to have TOP was easy as ‘there was not a single part of the child that is healthy’ (UK9, female, first child, chose TOP). Another UK parent described accepting that the physical features of the anomaly, despite a lack of diagnosis, were likely to indicate a poor prognosis. She went on to describe that she ‘knew then within myself that I wanted to terminate the pregnancy because he was just so sick’ (UK3, female, first child, chose TOP).

##### Role of healthcare professionals

###### Communication style

Many parents had positive experiences of the way HCPs communicated information to them, which they described as helpful when faced with uncertainty. Many parents both in the UK and the NL described how clinicians took their time speaking with them and answering any questions that they had. One UK parent explained how her clinician ‘didn't rush us through the actual findings’ and was ‘very good to answer our questions’ (UK6, female, first child, chose TOP), while a Dutch parent described how her clinician answered her questions ‘very well and very calmly’ which helped to put her at ease (NL4, female, pregnant at interview with first child, continued with pregnancy). Another UK parent reflected on the empathic manner of one clinician who made the effort to ‘normalize’ what was a difficult pregnancy and emphasized the love and care the baby would receive: ‘He really sort of spent time to talk about that, ‘look, you know, we'll care for your child, we'll love her as you would any other child’ and kind of injected that sense of normality too, that this is a normal pregnancy’ (UK1, partner, first child, continued pregnancy).

The empathic manner of clinicians was again highlighted when parents had to make a decision regarding TOP. One UK participant described feeling very cared for by her midwife when she arrived at the decision to terminate her pregnancy, further stating ‘I felt very recognised, I didn't feel judged [by her]’ (UK3, female, first child, chose TOP). Having an empathic, patient and caring approach was viewed as important by many when faced with uncertainty, with one Dutch parent commenting that: ‘with a little more empathy’ clinicians can ‘better guide people in dealing with uncertainty’ (NL4, female, pregnant at interview with first child, continued pregnancy).

###### Additional clinical support

A number of parents discussed how their clinician would provide extra clinical support which could help provide some comfort. For example, one UK parent described having the baby's heart rate monitored three times a week from week 22 of her pregnancy until her planned cesarean section (UK7, female, second child, continued pregnancy). Interestingly, one Dutch participant recalled how her obstetrician ‘stopped by’ her house if there was any new information to discuss (NL8, female, first child, chose TOP). This regular monitoring offered by clinicians helped to provide parents with some reassurance.

Some parents also spoke positively about how their clinician prepared them for the upcoming birth, particularly if the birth was likely to be complicated. At a time when parents were often given limited information about the anomaly identified, this was one way of obtaining some certainty about the process of giving birth and what could happen after. One UK parent explained that her clinician prepared her for the possibility of a cesarean section if they felt that the baby's heart was ‘under stress’ (UK7, female, second child, continued pregnancy). Some clinicians also provided support by showing parents the ward that they and the baby would be staying in; ‘The doctor made the effort to actually give us a tour of the ward that we'd be on, and like where she [baby] would be and where I might be, where the labor ward was’. (UK2, female, first child, continued pregnancy).

## 4 DISCUSSION

This is the first study to apply Han's taxonomy of medical uncertainty (Han et al., 2011) to a prenatal setting, specifically following the identification of a fetal anomaly after a routine ultrasound scan. Understanding the different dimensions of uncertainty that may result as a consequence of prenatal screening and testing are useful to guide HCPs in how they can best care for parents. The application of Han's taxonomy provided a useful framework for understanding prospective parents’ experience of uncertainty when faced with ultrasound anomalies and unclear findings from diagnostic tests. Our interviews with parents revealed examples of the different types of uncertainty described in the taxonomy regarding where uncertainty arises from (sources) and in relation to what the uncertainty was about (issues) (Table 3). Detailed understanding of the different dimensions of uncertainty that may arise as a consequence of prenatal testing and screening is useful for supporting parents and a summary of good practice points for HCPs working with parents who face uncertainty is presented in Table 4.

**TABLE 3:** Summary of findings using the Han Taxonomy as an interpretive framework

Dimension	Examples of application in the prenatal testing context
Source	
Probability	Likelihood that the anomaly on the scan could be a genetic issue There are a range of possible outcomes for the child
Ambiguity	Limited information is available about the anomaly Limited information regarding prognosis for the baby
Complexity	Complex information Complex explanations and use of medical jargon
Issue	
Scientific	Probable diagnoses with no further information (diagnostic) Further diagnostic tests unable to provide further information regarding potential treatment (therapeutic) Etiology of the anomaly (causal) Limited information regarding prognosis for the baby (prognostic)
Personal	Emotional impact of uncertainty—sadness, loneliness, self‐blame, grief Concern for future pregnancies—repeat of ‘history’ Anxiety post‐birth, waiting for ‘signs’ or ‘symptoms’ of anomaly to appear
Practical	Limited or unclear information about procedures, including where tests may take place and regarding termination of pregnancy. Limited information of what will happen following birth, for example, where the baby will be cared for

**TABLE 4:** Summary of recommendations for HCPs working with parents who face uncertainty in the setting of prenatal testing and screening

Good practice points	Dimension(s) of uncertainty addressed
Provide clear and simple information about medical procedures.	Source: Complexity Issue: Procedural
When discussing possible diagnoses, break up or simplify the information.	Source: Probabilistic, complexity, ambiguity Issue: Scientific and personal
Establish what parents know and understand prior to starting a discussion. Check that parents have a clear understanding of the information that they have been provided.	Source: Ambiguity, complexity Issues: Scientific, personal
Provide clear information about practical aspects of care, such as where procedures will take place and timelines, what will happen before, during and after procedures to allow parents a sense of certainty when so much else is uncertain	Source: Ambiguity, complexity Issue: practical
Be clear with setting expectations and be honest about uncertainty	Source: Probabilistic, ambiguity, complexity Issue: Personal
Allow parents time for discussion and time to go away and think.	Issue: Personal
Provide good written information, which can include carefully considered web links to descriptions of conditions, test procedures, further support.	Source: Ambiguity, complexity Issue: Practical, scientific, personal
Put families in touch with specialist health professionals, (e.g., psychologists, counselors) and support groups—early if possible.	Issue: Personal
Allow for flexibility in providing additional clinical support and/or regular monitoring which can provide additional reassurance to parents	Source: Ambiguity Issue: Scientific, practical, personal

Many of the experiences of uncertainty identified through this study are consistent with findings in the published literature (Harding, Hammond, Chitty, Hill, & Lewis, 2020). Regarding the ‘sources’ of uncertainty (*probability*, *ambiguity,* and *complexity*) we found, for example, that the uncertainty related to *ambiguity* included the limited information available to parents about the anomaly identified. In a study looking at pregnant women's experience after abnormal ultrasound findings, Asplin et al. found that the types or amount of information given to women contributed to feelings of uncertainty. This ambiguity subsequently led to women feeling that they had too little time for pregnancy‐related decision‐making (Asplin et al., 2012). In our study, uncertainty stemming from *complexity* included both the information itself and how it was communicated to parents, with the use of medicalized language seen as problematic. Similarly, Walser et al. found that parents receiving prenatal CMA results reported difficulties understanding some of the terminology used by the HCP (Walser, Werner‐Lin, Russell, Wapner, & Bernhardt, 2016). Other studies have shown that patients, even with adequate literacy, may have difficulties understanding what they are being told due to medical terminology and jargon (Rubel, Werner‐Lin, Barg, & Bernhardt, 2017).

The ‘issues’ of uncertainty identified here (*scientific*, *personal,* and *procedural*), included personal issues such as shock, anxiety, guilt and self‐blame, which have also been seen in other studies. For example, Werner‐Lin et al. found that couples receiving abnormal prenatal CMA results were ‘blind‐sided’ and unprepared to receive results for which clear risk estimates of diagnostic meaning were unknown (Werner‐Lin et al., 2016). We also found that anxiety resulting from uncertain findings from prenatal testing could continue after the birth. This is similar to research looking at mothers’ anxiety after receiving false‐positive results during new‐born screening, and heightened anxiety related to potential chronic illness later on in the child's development (Hayeems et al., 2016; Price Dillard & Carson, 2005). Other studies have also found that parents who had received uncertain prenatal test results remained worried and uncertain about their child's development after the birth (Bernhardt et al., 2013; Desai et al., 2018). Werner‐Lin et al. noted heightened vigilance in mothers whose children were prenatally diagnosed with a copy number variant of uncertain significance (Werner‐Lin et al., 2016). In our study, parents also reported experiences of *procedural* issues of uncertainty and expressed that there was *ambiguity* surrounding the information available to them for example, regarding timelines to make a decision about medical procedures, information about procedures such as termination, and other practical aspects of care such as where the baby would be born and which ward or unit they could be moved to. This is consistent with findings from other studies that have highlighted the importance of clear information regarding medical procedures, including next steps (Asplin et al., 2012; Black & Sandelowski, 2010). Limiting the ambiguity surrounding the diagnostic process and practical aspects of care could be beneficial in helping parents to cope with diagnostic uncertainty.

When faced with hearing difficult and complex news, and what may happen going forwards, HCPs should be mindful about how information is delivered to parents, and establish what parents know and understand prior to starting a discussion (Biesecker et al., 2014).

Parents’ desire and need for support to manage uncertainty in this setting is not uncommon (Bernhardt et al., 2013), and through this work, we were able to identify the strategies parents used for managing their uncertainty, many of which have been previously documented in the literature (Walser et al., 2016; Werner‐Lin et al., 2016). Parents used practical strategies as well as internal and external coping strategies to manage different elements of uncertainty. For example, information seeking was a practical strategy to help try and bring clarity to *complex* or *ambiguous* information, such as those related to probable diagnoses or medicalized language (*sources* of uncertainty). Information seeking through Internet searches was a common approach used by parents to try and obtain information that has been seen in other studies (Denney‐Koelsch, Côté‐Arsenault, & Lemcke‐Berno, 2015; Lotto, Smith, & Armstrong, 2018). Internal coping strategies used included ‘remaining hopeful’ and making a conscious decision not to worry or ruminate over the uncertainty, a coping mechanism also found in a recent study conducted with pregnant women receiving uncertain CMA results in Denmark (Lou et al., 2020). Some parents had also used ‘positive reappraisal’ as a method to cope with the difficulty of the decision to end their pregnancy. Such adaptive coping strategies to manage emotional distress are well illustrated in the literature to manage the grief that can be felt when ending a pregnancy due to a fetal anomaly (Lafarge, Mitchell, & Fox, 2013). External coping strategies included seeking social support from family and friends, as well as support groups or charities. Having a social network, whether these are people that have had similar experiences or not, can help parents navigating uncertainty in the prenatal setting whether this is through providing reassurance or sharing experiences with others (Rubel et al., 2017).

For some parents in this study, HCPs provided emotional social support, particularly during difficult circumstances such as when having to consider TOP. Studies have reported the benefits of having HCPs to provide social support during this complex time (Bratt, Järvholm, Ekman‐Joelsson, Mattson, & Mellander, 2015; Edvardsson et al., 2014). However, this is not always easily accessible or possible. Having access to social or psychological support was viewed as beneficial by parents. Some parents felt their referral to professional support came too late in their journey, and some had to seek out their own supportive networks, for example, through social media. Therefore, in addition to providing information about what support is available, timely referrals or directing to specialist counselors, charities or groups would be beneficial in optimizing psychological support to parents who receive unexpected news and who may have to make difficult decisions regarding their pregnancy.

Overall, we found that HCPs communication style and the type of support provided, either emotional or more practical support (such as giving parents a tour of the ICU), influenced how parents responded to and managed uncertainty. Other studies have highlighted that HCP communication has an overarching impact on the parent's experience of receiving a prenatal diagnosis for their unborn child. In particular, how the information is communicated was thought to affect parent's ability to cope with uncertainty (Bratt et al., 2015). Preparing parents for the possibility that uncertainty may occur may help patients become more tolerant of uncertainty, though how this could be achieved and what type of ‘preparatory discussion’ works best needs further research. Biesecker et al. goes further and suggests that examining factors such as patients’ tolerance for uncertainty, resilience, and optimism in conjunction with patients’ expectations about genomic testing may help identify those more likely to appraise uncertainty as a threat and to mitigate negative responses (Biesecker et al., 2014).

### 4.1 Strengths and limitations

A key strength of this study is that by applying the Han taxonomy to the uncertainties that follow on from receiving an abnormal fetal anomaly scan, we have been able to delineate the multiple forms of uncertainty that occur for parents. This in turn has provided a more nuanced understanding of the nature of uncertainty in the prenatal setting. Though the UK and the NL have different healthcare systems and differences in cultures, this study found that parents from both countries experienced similar types of uncertainty that mapped onto each dimension of the taxonomy. While this could suggest that the experiences of parents remain similar regardless of the country they reside in and differences in prenatal care, this may have been influenced by the sample size and selective recruitment strategy for this study. Finally, applying this taxonomy to a population of parents from the UK and the NL demonstrates the generalizability of this taxonomy of uncertainty in health care. In agreement with Han and colleagues, this taxonomy facilitates an organized approach to understanding the issue of uncertainty in health care, its unique nuances and aids in suggesting appropriate strategies for its analysis and management (Han et al., 2011).

In future research, application of Han's taxonomy of genomic uncertainty (Han et al., 2017) may provide useful insights when specifically exploring patient's experiences of receiving prenatal ES results.

The study has a number of limitations. Due to the way parents were recruited, there were differences and biases between the populations. Only Dutch participants underwent ES and it is therefore possible that for Dutch parents, receiving ES results may have had a significant influence on their overall perceptions of uncertainty. All but two parents from the UK were recruited from a parent support group (ARC). It is possible that these parents had particularly negative experiences that led to them seeking support. The timeframe of uncertainty was anywhere from one to 11 years ago in the UK, with all Dutch parents experiencing uncertainty up to a year before they were interviewed. This may have affected recall of their experience, and additionally, health practices may have changed significantly in that time. Only one father volunteered to be interviewed, and the experience of uncertainty and the application of this to the taxonomy is largely based on female parents’ experience of uncertainty. Therefore, these results are not transferable to fathers. Additionally, the small sample size, although does provide some insight, does make it difficult to draw general conclusions and provide an in‐depth insight into parents’ experiences of uncertainty. Finally, most of the parents in this sample were highly educated; participants with lower educational attainment may experience uncertainty differently.

## 5 CONCLUSIONS

The findings from this study have provided novel insights into the way uncertainty is experienced in the prenatal setting, and the importance for parents of having clear information regarding the practical aspects of both medical procedures and care. Going forwards, we must be mindful of the likely increase in uncertain results that may arise from next‐generation sequencing and how this may affect parents who are already in a state of uncertainty. By ensuring that communication is clear and parents are as prepared as possible, with adequate psychological support provided, we can try to minimize the negative impact of the experience of uncertainty in the prenatal setting.

## AUTHORS’ CONTRIBUTIONS

CL conceived the study. All the authors inputted into study design and best practices for data acquisition. JH and JEK developed the codebook and coded the interviews. JH, JEK, MH, and CL analyzed the data. All the authors were involved in data interpretation. JH and JEK drafted the paper. All the authors revised the paper critically for important intellectual content and have approved the paper for publication.

## COMPLIANCE WITH ETHICAL STANDARDS

In the UK, ethical approval for this study was obtained through London—Riverside Research Ethics Committee (18/LO/2120). In the Netherlands, ethical approval was obtained through the Erasmus Medical Centre Medical Ethics Review Committee.

### Conflict of interest

The authors do not have a conflict of interest.

### Human studies and informed consent

This study was reviewed and approved by London—Riverside Research Ethics Committee (18/LO/2120). In the Netherlands, ethical approval was reviewed and obtained through the Erasmus Medical Centre Medical Ethics Review Committee. All procedures followed were in accordance with the Helsinki Declaration of 2000. All participants gave informed consent to participate prior to interview.

### Animal studies

No non‐human animal studies were carried out by the authors for this article.

## Supporting information

Supplementary Material

## Data Availability

Consent was not given to publish interviews in full and online. However, portions of the data are available from the corresponding author on reasonable request.
